# Identifying unmet needs from the General Medical Council Gateways to the Professions guidance

**DOI:** 10.15694/mep.2018.0000057.1

**Published:** 2018-03-07

**Authors:** Miriam Antcliffe, Francis Leng, Ioanna Maraki

**Affiliations:** 1 The Right to Know Ltd.; 2 General Medical Council

**Keywords:** Disabilities, Medical Education, Equal Opportunity (Education), Educational Guidance

## Abstract

This article was migrated. The article was marked as recommended.

The General Medical Council (GMC) commissioned The RTK Ltd to conduct a research project to inform a revision of the *Gateways to the Professions* guidance *(The Guidance). The Guidance* supports medical education providers to ensure that disabled students and doctors in training (disabled learners) do not face unnecessary barriers to successful medical careers. The researchers consulted with medical schools, HEE Local Teams, deaneries and employers on their use of the current guidance and good practice. They also interviewed medical students to better understand their experiences of admissions procedures and the support they were able to access during their period of study. It was evident that the provision for disabled learners is varied; the research found no single established model of support. However, the interviews revealed some key principles of good practice and innovative suggestions for future provision. Medical education providers perceived a need for a revised version of *The Guidance* to reflect good practice and provide more specific advice to them as well as to leaners.

## Introduction

The General Medical Council (GMC) are responsible for overseeing all stages of medical education in the UK, and have a commitment to ensure that fairness is embedded throughout. In 2008, the GMC launched the *Gateways to the professions* guidance ( *The Guidance). The Guidance* provides practical suggestions to education providers on supporting disabled students and doctors in training. *The Guidance* was most recently updated in 2013.

The GMC commissioned a social research company the RTK Ltd to gather evidence to inform a new revision of the *Gateways* guidance.

Specifically, the current project set out to gather:

1. evidence from medical schools, providers of postgraduate medical education (HEE local teams and deaneries) and employers on how they currently use *The Guidance*, examples of what they consider to be good practice, any challenges posed by the existing *Guidance* and suggestions about what would be useful in the revised guidance; and
2. the views of medical students concerning their experiences of admissions procedures, and the support available during their period of study.

The research was shaped by the social model of disability. The social model was developed as a challenge to what is known as the medical model of disability and has been adopted by organisations such as the World Health Organisation and the UK Council for Disabled People. The social model distinguishes between the terms impairment and disability; whilst impairment refers to a physical or psychological attribute or condition, disability refers to the range of social, economic and physical barriers experienced by people with impairments.

## Methods

The approach to the research was to use a mixed methods design that is summarised in Figure 1 below.

**Figure:**
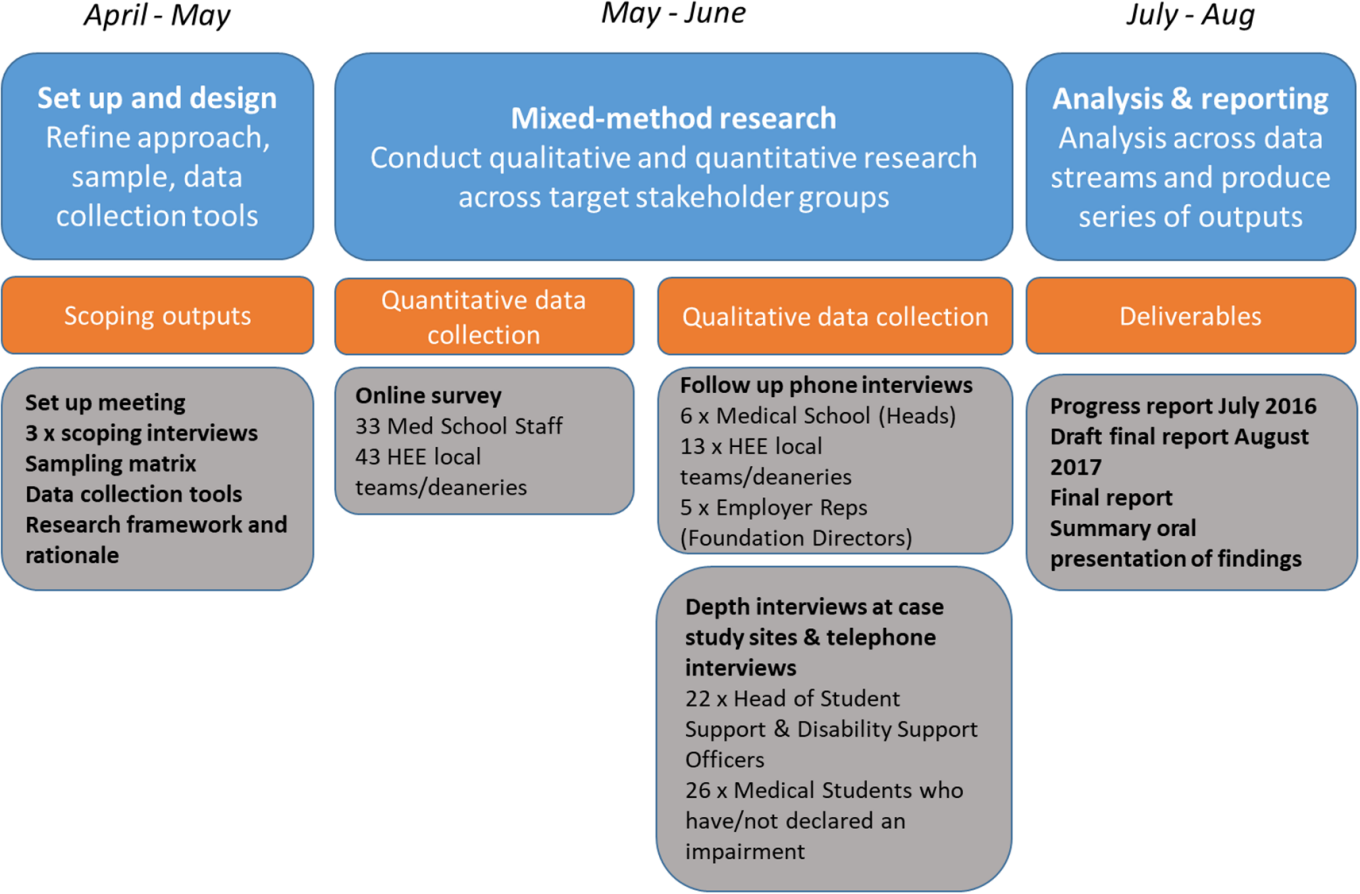


The scoping phase involved three interviews with key strategic stakeholders. These insights were used to inform the development of the fieldwork tools:

• An online survey for stakeholders across undergraduate and postgraduate medical education providers. The purpose of this was to gather information about how *The Guidance* is currently used by education providers and the barriers and enablers to its effective use.
• Qualitative interview topic guides. Separate guides were developed for:
  • Disabled Medical students - designed to explore their experiences of the admissions process, any barriers they faced whilst on the course either at university or on placement, and their experiences of seeking support through the medical school.
  • Heads of medical schools, HEE local teams/deaneries and employer representatives - designed to develop a high level understanding of how *The Guidance* is used and understand the systems in place to support learners.
  • Head of Disability or Student Support roles - designed to get a view of how disabled learners are supported, key challenges to doing so, aspects of practice that work well, and their views on what is needed from the revised guidance.

Fieldwork took place from May to July 2017. We conducted an online survey of 76 stakeholders across providers of undergraduate and postgraduate medical education. We went on to complete face to face and telephone interviews with 46 of these stakeholders, including foundation school directors, deans of HEE local teams/deaneries, heads of medical schools, and disability/welfare officers. We also interviewed 26 disabled medical students (including two student representatives) with a range of impairments.

The research team met for an analysis workshop, debating the key themes and trends from across the stakeholder groups. We developed a shared analysis framework organised around these key themes to manage the qualitative data and facilitate a robust thematic analysis. The approach to analysis enabled us to identify the key areas where the views of the stakeholders we spoke to aligned, whilst also allowing us to explore the often more nuanced points where they diverged. What was particularly striking was the broad level of agreement across stakeholder groups, including students themselves, about the general principles of good practice.

## Results

### Current Use of the *Gateways to the professions guidance*

The responses to our online survey indicate that awareness of *The Guidance* is far more common among undergraduate medical education providers (88% of those surveyed) than among postgraduate education providers (48% of those surveyed). For those that were aware of it, the results of the survey indicate that *The Guidance* is not currently used on a regular basis or referred to directly for each individual case.

**Table:** 

Medical schools	Postgraduate education providers
1. Understanding responsibilities of the organisation (82% of those surveyed)	1. Understanding responsibilities of the organisation (83% of those surveyed)
2. Considering requests for reasonable adjustments (73% of those surveyed)	2. Informing doctors in training of the support that can be provided to them (67% of those surveyed)
3. Informing internal procedures (63% of those surveyed)	3. Providing technical assistance and equipment (67% of those surveyed)

These results suggest that *The Guidance* is used primarily to understand the responsibilities of the organisation towards disabled learners. It is also useful for setting the tone and ethos of their work and helping them to understand their legal duties. Education providers used *The Guidance* much less frequently as a guide on how to address specific issues such as considering individual requests for reasonable adjustments and encouraging disabled learners to disclose their impairment.

### Current provision of support and attitudes towards disability in medical education

There is no single model of provision for disabled learners in medical education. At undergraduate level some medical schools have standard processes that may include assigning caseworkers to students and engaging panels of experts in the decision making process. Other medical schools may only have a few individuals who make these decisions on a more ad hoc basis. Additionally, while some medical schools work closely with university-wide services, others are totally independent. Interviews with the deans and vice deans of HEE local teams and deaneries suggested that the processes for disabled doctors are less standardised at postgraduate level and that there is no one individual with responsibility for supporting disabled doctors.

The research revealed different cultures and attitudes towards disability. Education providers generally expressed a view that progressive, more inclusive attitudes were becoming more prevalent. However, providers commonly remarked that whilst flexibility within study is very achievable; when it comes to the high pressured work environment of the NHS, this is not always possible.

### The experience of students

The self-selecting sample of medical students we spoke to were all at different stages of study. Our interviews revealed that students can have concerns about disclosing an impairment to the medical school because they are unaware that they would be considered disabled or are unsure about what support could be offered. Students were also concerned that disclosing a disability could have future fitness to practise implications.

*“I was concerned about confidentiality, whether it would impact on future job applications, whether it would go in my medical notes, that kind of thing, particularly with mental health - you feel judged.” - Student*

In medical schools where students are encouraged to disclose, they seemed more likely to at an earlier stage and less worried about the implications of doing so. Most of the students reported that once they had disclosed an impairment, they were reassured by the level of support they received from their medical school. The students we spoke to had low awareness of the legal responsibilities providers have towards them during education and employment, they were keen that students have access to the revised guidance so they are better informed about the support they could access.

Students’ experiences were variable, but even the students who reported generally positive experiences had faced barriers at some stage. Those barriers fell into five categories:

**Communication/information barriers:** typically, this was when communication broke down between the many different stakeholders involved in medical education such as between the medical school and the placement provider.

**Physical barriers:** on the whole university campuses were mostly accessible for the students we spoke to. However, physical barriers were more often encountered on placement. Difficulties travelling to placements, challenges moving around wards and hospitals and dealing with loud background noise were all examples of physical barriers faced by students.

*“I missed out on learning opportunities on placement because my wheelchair didn’t always fit on the ward.” - Student*

**Financial barriers:** only a minority of students described situations when either they could not finance equipment or they experienced a lack of resources. However, the consequences when such situations occurred were far reaching.

**Cultural barriers:** typically, we found that positive cultures and attitudes were commonplace at medical schools. However, students made a distinction between this and the profession as a whole. They were acutely aware of the pressures facing the NHS and felt that this would impact on the level of care and consideration they could expect to receive in their future career. Students identified a culture of perfectionism in medicine as a factor contributing to these barriers, a culture they acknowledged they had a role in perpetuating.

*“There is a view that you have to be superhuman and can’t get as much as a cold, got to always be 100%” -* Student

**Experiences of overt discrimination:** in a small number of cases students described how negative encounters with individuals had made them more reticent about seeking support in the future. These rare examples of overt discrimination experienced by students all happened when they were on placement.

Many of the students described much more positive experiences. Crucial relationships with staff, tailored support, and collaborative decision making were all common themes. The students often identified flexibility and effective communication as being vital components in overcoming barriers to medical education.

*“I explained my condition and they explained what would be expected in the course, we identified barriers and solutions together, it was collaborative” - Student*

### Challenges in supporting disabled learners

Ensuring that support was implemented at both undergraduate and postgraduate levels was not without challenges. This was often due to a breakdown in communication between the education provider and the placement or employment provider, further complicated by issues of confidentiality. At postgraduate level, a number of the deans and vice deans we spoke to felt that the Transfer of Information (TOI) process was not happening efficiently enough and could hinder their ability to prepare adequately. At undergraduate level, tensions between departments could occur when university-wide disability or occupational health services made recommendations that, in the view of the medical school, were not applicable to medical training.

*“There is an advocacy role because sometimes placements are unhelpful and will not manage the environment, we can’t force them to take doctors in training but we have to advocate making the adjustments, particularly small trusts who haven’t come across these issues before are reluctant so you get to know people in trusts who are more helpful.”- Postgraduate Dean*

A common concern was a perceived paradox that by providing support and adjustments, medical educators could in fact be setting students up to fail in the long term as this level of support may be unavailable in a workplace. Some providers also expressed frustration with what they felt was a lack of flexibility in course requirements, particularly the clinical competencies. In their view this prevented otherwise talented doctors from progressing, even if they were unlikely to use that skill again.

In the extremely rare circumstances of learners not being able to complete courses, interviewees described that it was usually due to compounding factors of which the individual’s health status was only one. It was evident that these were extremely difficult decisions to take for all those involved.

### Good practice in supporting disabled learners

Despite the changing nature of medical education at undergraduate level through to postgraduate level, the key elements of good practice remained largely consistent. The elements of good practice identified by providers largely mirrored those identified by students.

Providers made reference to the importance of fostering a supportive culture towards disability in which students and doctors feel able to be open about seeking support. It is critical to establish this culture of openness and inclusivity before learners start their programme of study, as early disclosure allows for a planned and proactive response to individual needs. Providers who were demonstrating good practice had used *The Guidance* to shape their ethos and establish clear internal procedures.

*“I had email contact with the university before I started, I sent over my report of the assessment that I had had from an independent disability assist service after finishing my a-levels, they agreed to implement everything on my needs assessment” - Student*

Good practice meant that providers were assured that a range of options had been considered and experts, as well as the student themselves, had been consulted. This meant that generally all stakeholders were satisfied, and in instances where this was not the case there were procedures for it to be rectified. Regular monitoring and review were key, particularly for those students whose needs were changeable due to the nature of their impairment and the course requirements.

Effective communication between all parties was also vital to good practice. For universities this was particularly successful when they sought close collaboration with occupational health departments or university-wide disability services; and for postgraduate providers when they fostered good working relationships with employers.

*“We have very close links between the school of medicine support and the university support services. Close communication with the experts in the DDS [Disability and Dyslexia Service]are essential to ensure the correct adjustments are provided.” Head of Student Support*

Another hallmark of good practice was provision of support tailored to the individual learner. This commonly resulted from comprehensive needs assessments undertaken by a personal adviser in partnership with a learner to consider holistic solutions to a number of barriers. For example at postgraduate level this could involve carefully selecting placements and providing individual, specialised careers advice. Interestingly, some providers also mentioned a move towards ‘universal adjustments’, proactively changing the environment to make it accessible for all. Adjustments to the colour of paper and font style/size on all written materials were examples we came across.

Providers who prioritised issues of disability were keen to ensure that these services continued to improve and were fit for purpose. They also felt it was critical that their organisations adopted a culture of learning; ongoing training and peer workshops were highlighted as important elements of sharing good practice.

## Discussion

The results from interviews and the survey clearly indicated that providers of both undergraduate (82% of those surveyed) and postgraduate (67% of those surveyed) medical education thought that the current guidance needed updating. One priority was for there to be a clear, shared understanding of definitions of disability and clarity around which learners require support. It was felt that this could be addressed more thoroughly in the updated guidance.

Providers wanted clearer parameters about what level of adjustments should be considered to be ‘reasonable’ and better consistency of practice. However, providers accepted that guidance cannot provide simple, formulaic solutions. Some suggested that the new guidance could inform processes including who should be involved in decision making, how to maximise the expertise of specialists such as occupational health and what needs to be considered in order to create robust procedures.

*“Some sort of framework of questions to ask to interrogate your processes and to evaluate and improve the process. We can’t have a one size fits all approach but we could have a best practice process which we can all aim for.”- Head of Medical School*

Both students and providers felt that it would be useful for students to have access to the revised guidance from pre-application stage. That way they would be better informed about the level of support they could expect and consider potential barriers. Providers also felt that the revised guidance should support them in having frank and honest discussions with students in the rare situations where learners faced barriers that meant they were unlikely to progress despite all possible support being offered.

The findings from the research will inform the revision of the guidance as part of a wider programme of work by the GMC, looking at how disabled students and doctors are supported throughout medical education to improve access to the profession.

The core aim of the work programme is to provide more practical advice to medical education providers, building on the overriding principles of the existing *Gateways to the professions* and equality legislation, and expanding advice given for postgraduate training. The barriers and principles of good practice highlighted by the research are going to be reflected as part of the suggestions given within the revised guidance. Another aspect of the advice will be stepwise frameworks to help decision-making. Specifically, on postgraduate training, the revised guidance will highlight existing mechanisms that can be used for supporting disabled doctors.

The revised guidance will also discuss the importance of sharing information to ensure the right support is in place for disabled learners and reiterate the GMC’s position that having a health condition or disability is not a fitness to practise issue.

Another aim of the work programme is to reach doctors and students directly, to increase awareness of the guidance. This will be done in collaboration with the Medical Schools Council for communication with medical students.

The guidance will be accompanied by a set of resources in order to produce a ‘hub’ of helpful materials for medical students, doctors and educators. These will include published research on the access of disabled people into the medical profession, short films and personal stories from disabled students and doctors.

## Research ethics

The RTK is a social research company made up of academics and practitioners. As such, the RTK adheres to ethical guidelines from the Social Research Association, British Association of Social Workers and the British Psychological Society. During this project the RTK produced an ethical statement detailing the purpose and methods of the research, action that would be taken to remove barriers to participation as well as the steps that would be in place to avoid any potential harm to research participants. Participation in the research was based on voluntary and informed consent. All information was stored in accordance with the Data Protection Act 1988 and the RTK is registered with the Information Commissioner’s office.

## Take Home Messages

• Undergraduate education providers were more familiar with the *Gateways to the professions* guidance than postgraduate providers. Three quarters of those surveyed thought the current guidance should be updated.
• Current practices for supporting students and doctors are variable. There isn’t a single process followed by medical schools or postgraduate providers, although similarities exist.
• Students sometimes did not share information about their health because they were unsure what support would be offered and were worried about fitness to practise implications.
• Disabled students encounter different types of barriers, including communication, physical, financial, and cultural barriers. Students would like to be better informed about the support that is available to them and for any form of revised guidance to be shared with them.
• The findings outline key principles for supporting disabled students and doctors in training. These include fostering a positive culture; having clear established processes and supporting effective information sharing.
• Providers said they wanted the revised guidance to include assurance for decision-making processes, and tools for quickly accessing and interacting with the content.
• The findings from the research will inform the revision of the guidance as part of a wider programme of work by the GMC, looking at how disabled students and doctors are supported throughout medical education to improve access to the profession.

## Notes On Contributors

Miriam Antcliffe is the Research Director for the RTK Ltd, a research consultancy. Miriam is a highly experienced social researcher and is also a qualified social worker with a background in children and families practice. She has a particular interest in ensuring research findings are translated and embedded into practice.

Francis Leng is a research officer at the General Medical Council (GMC). He manages internal and externally commissioned GMC research projects. Before moving to the GMC, Francis spent two years as a research and insight advisor for the English Federation of Disability Sport.

Ioanna Maraki is an Education Policy Manager at the General Medical Council (GMC). She is currently working on the revision of the GMC’s *Gateways to the professions* guidance on supporting disabled medical students and doctors. Ioanna holds a BSc in Biomedical Sciences from the University of Edinburgh and is completing a MSc in Global Health Policy at the London School of Hygiene and Tropical Medicine.
