# Supplementary material for: Identifying unmet needs from the General Medical Council Gateways to the Professions guidance
Source: MedEdPublish (2016). 2018 Mar 7;7:57. [Version 1] doi: 10.15694/mep.2018.0000057.1 (PMC10711940; doi:10.15694/mep.2018.0000057.1)
Supplement: Peer review history

# Reviewer report: Reviewer response for version 1

**DOI:** 10.21956/mep.19524.r28893

**Published:** 2018-03-26

**Authors:** Janusz Janczukowicz

**Affiliations:** 1 Medical University of Lodz

This review has been migrated. The reviewer awarded 4 stars out of 5 This paper presents a research project focused on evaluation of current GMC guidance regarding ensuring that disabled learners do not face unnecessary barriers to their careers. While this study is focused on a local UK contexts, its multiple findings are highly transferable to the other regions, including the complexity of barriers faced by disabled learners and students’ worries regarding their fitness to practice enhancing their reluctance to share information about their health. This publication should enhance reflection of all stakeholders of medical education regarding the disabled learners access to the profession.

Reviewer Expertise:

NA

No decision status is available

# Reviewer report: Reviewer response for version 1

**DOI:** 10.21956/mep.19524.r28892

**Published:** 2018-03-09

**Authors:** Judy McKimm

**Affiliations:** 1 Swansea University

This review has been migrated. The reviewer awarded 4 stars out of 5 This is an interesting study which i enjoyed reading. It highlights a range of issues as well as offers useful ways forward for UG and PG educators and support staff as well as for the UK regulator, which is very welcome and overdue. There is still a lot of stigma and lack of understanding from both learners and educators as to how best to address issues around disability, and some common myths which this article starts to dispel. Many thanks for undertaking the research and I look forward to the new guidance!

Reviewer Expertise:

NA

No decision status is available
